# *FBXW7* mutations typically found in human cancers are distinct from null alleles and disrupt lung development

**DOI:** 10.1002/path.2874

**Published:** 2011-04-18

**Authors:** Hayley Davis, Annabelle Lewis, Bradley Spencer-Dene, Hilda Tateossian, Gordon Stamp, Axel Behrens, Ian Tomlinson

**Affiliations:** 1 Molecular and Population Genetics Laboratory, Wellcome Trust Centre for Human Genetics, Oxford UniversityRoosevelt Drive, Oxford OX3 7BN, UK; 2 Experimental Histopathology Laboratory, London Research Institute, Cancer Research UK44 Lincoln's Inn Fields, London WC2A 3LY, UK; 3 MRC Mammalian Genetics UnitHarwell, OX11 ORD, UK; 4 Mammalian Genetics Laboratory, London Research Institute, Cancer Research UK44 Lincoln's Inn Fields, London WC2A 3LY, UK

**Keywords:** Fbxw7, lung development, TGF-β, WD40, cancer

## Abstract

FBXW7 is the substrate recognition component of a SCF-type E3 ubiquitin ligase. It has multiple targets such as Notch1, c-Jun, and cyclin E that function in critical developmental and signalling pathways. Mutations in *FBXW7* are often found in many types of cancer. In most cases, these mutations do not inactivate the protein, but are mono-allelic missense changes at specific arginine resides involved in substrate binding. We have hypothesized that *FBXW7* mutations are selected in cancers for reasons other than haploinsufficiency or full loss-of-function. Given that the existing mutant *Fbxw7* mice carry null alleles, we created a mouse model carrying one of the commonly occurring point mutations (*Fbxw7*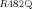) in the WD40 substrate recognition domain of Fbxw7. Mice heterozygous for this mutation apparently developed normally *in utero*, died perinatally due to a defect in lung development, and in some cases showed cleft palate and eyelid fusion defects. By comparison, *Fbxw7*^+/−^ mice were viable and developed normally. *Fbxw7*^−/−^ animals died of vascular abnormalities at E10.5. We screened known FBXW7 targets for changes in the lungs of the *Fbxw7*^*R*482*Q*/+^ mice and found Tgif1 and Klf5 to be up-regulated. *Fbxw7*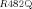 alleles are not functionally equivalent to heterozygous or homozygous null alleles, and we propose that they are selected in tumourigenesis because they cause a selective or partial loss of FBXW7 function. Copyright © 2011 Pathological Society of Great Britain and Ireland. Published by John Wiley & Sons, Ltd.

## Introduction

*FBXW7* (*FBW7, CDC4*) is a tumour suppressor gene on human chromosome 4q that encodes the substrate recognition component of an E3 SCF-type ubiquitin ligase complex. This specific E3 ligase complex negatively regulates the abundance of an expanding list of key proteins such as CCNE1, JUN, MYC, NOTCH1, AURKA, KLF5, and TGIF1. It is currently unclear whether some or all of the substrates are regulated in a tissue-specific and/or developmental manner. However, Nicd1 (active notch1) and c-Jun appear to be key substrates in mouse embryonic fibroblasts 1, 2, c-Myc in haematopoietic stem cells 3, and Notch and c-Jun in the intestine 4. There are several excellent reviews of the growing knowledge of FBXW7 5–7. Substrates of FBXW7 contain a consensus sequence called a phosphodegron 8. FBXW7 recognizes phosphorylated phosphodegrons and thus targets these substrates for degradation by the proteosome. FBXW7 contains an N-terminal F-box domain and a C-terminal protein-interacting domain made up of eight WD40 repeats. These propellers provide a binding pocket for the substrates, and the apices of these propellers, especially critical arginine residues, are vital in substrate recognition. Different residues in the propellers are involved in ubiquitin binding 9 and hence the autoregulation of FBXW7 levels.

It has been proposed that *FBXW7* is a tumour suppressor gene (TSG) 2 and mutations occur at a moderate frequency in cancers of several anatomical sites, including the colorectum, stomach, blood, bile duct, and endometrium 10. However, we previously noted that the *FBXW7* mutation spectrum is not typical of a TSG 11. This view is confirmed by mutation data from the Sanger Institute Catalogue Of Somatic Mutations In Cancer (http://www.sanger.ac.uk/cosmic), which shows most mutations to be mono-allelic with the ‘second hits’ typical of a TSG occurring rarely 12. Furthermore, it is not clear that the most commonly occurring mutations—missense changes at the arginine residues at the tips of the substrate-binding propeller blades—result in loss of protein function, and only about 15% of mutations are predicted to lead to a truncated FBXW7 protein. One possibility is that the arginine propeller tip mutations are haploinsufficient or abolish FBXW7 function by acting as dominant negatives 10, 13. However, the unusual mutation spectrum at *FBXW7* in human cancers was reminiscent of the adenomatous polyposis coli (*APC*) gene, in which cancer-associated mutations are selected for partial retention of protein function 14 so as to provide a “just right” level of Wnt signalling 15. We therefore speculated whether the arginine propeller tip mutations in *FBXW7* also provide a specific selective advantage for tumourigenesis. In order to test this hypothesis, we wished to construct a suitable *Fbxw7*-mutant mouse to search for phenotypic and functional differences from null alleles.

According to the genetic data, where few cases in which *in-trans* truncating and missense mutations occur, simple haploinsufficency cannot explain the mutation spectrum 16. WD40 missense mutations may cause a selective loss of function (for example, of particular substrates only or in a tissue-specific fashion) or a partial loss of function (whereby mutant FBXW7 is not optimal in substrate degradation). In addition, because FBXW7 contains an N-terminal dimerization domain and can form dimers 13, it could also act as a dominant negative 10. Gain of function is not probable considering that the genetic data show that FBXW7 inactivating mutations occur at a low frequency.

Several *Fbxw7*-mutant mice have previously been generated 17, 18, but all of these carry simple or conditional null alleles. In the ubiquitous heterozygous mutant state, these mice appear normal, with no tumour formation up to 1 year of age, whereas in the homozygous state, the mutation causes embryonic lethality at stage E10.5 due to compromised vascular and cardiovascular development. Elevated levels of Notch 1 and 4, leading to stimulation of downstream pathways involving Hes1, have been observed 17, 18. Elevated cyclin E levels were found by Tetzlaff *et al* 17, but not Tsunematsu *et al*, the latter suggesting that Fbxw7 is dispensable for cyclin E degradation, at least until mid-embryogenesis 18. It is possible that cyclin E is regulated by other mechanisms until this time. Conditional null *Fbxw7* models have been used to assess its role in the haematopoietic 3, 19, 20 and gut lineages 4.

In order to test the specific effects of the arginine propeller tip mutations found in human cancers, we generated a mouse carrying an *Fbxw7*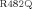 point mutation and compared our mice with existing null *Fbxw7* models.

## Materials and methods

### Generation and genotyping of R482Q mice

Mice were derived using standard methods; in brief, the target construct was cloned, linearized, and electroporated into 129Sv/J ES cells. Targeted ES cells were injected into C57Bl/6J blastocysts. The resultant chimeras were bred with C57Bl/6J mice for more than six generations. To identify homologous recombinants in the ES cells, the Roche Long Range Expand long template PCR system (Roche Applied Science, Basel, Switzerland) was used, followed by Southern blot using standard methods 21 (details available on request).

For genotyping PCRs, DNA was extracted from ear snips or embryo tails. Primers 1F (5′-TTCCTCACTTC CCATTCCAG-3′) and 3R (5′-TCTCTGGATCCCACA CCTTC-3′) were used to identify the floxed allele, and primers 1F and 6R (5′-GATTGGCCAGTACTGAACC T-3′) were used to identify the deleted allele.

### Mouse procedures

All procedures were carried out in accordance with Home Office UK regulations and the Animals (Scientific Procedures) Act 1986. All mice were housed at the animal unit at Clare Hall Laboratories, Cancer Research UK.

### Embryo collections

Gestation was dated by the detection of a vaginal plug (as E0.5). Embryos were dissected out of the womb and killed by decapitation. Various tissues were dissected and either snap-frozen in liquid nitrogen or fixed in 10% neutral buffered formalin (NBF).

### Histology

Specimens of 10% formalin-fixed tissue were embedded in paraffin and then sectioned at 4 µm. Sections were stained with H&E for histological examination following standard protocols. For coronal embryo head sections used to analyse cleft palate and EOB phenotype, the samples were decalcified in DFB (Pioneer Research Chemicals, Colchester, UK) solution for 48 h prior to processing.

### Sequencing

RNA was isolated from frozen tissue with the RNeasy minikit (Qiagen, Hilden, Germany) and treated with DNase I to degrade residual DNA, according to the manufacturer's instructions. Complementary DNA was reverse-transcribed *in vitro* from extracted RNAs, using the High Capacity cDNA Reverse Transcription Kit (Applied Biosystems, Foster City, CA, USA), and cDNA samples were purified with the QIAquick purification kit (Qiagen) following the manufacturer's instructions. Sequencing of cDNA to confirm the presence of mutation was carried out using the 2× Big Dye Terminator v3.1 reagent (Applied Biosystems). Purified products were run on the ABI 3730 DNA analyser (Applied Biosystems).

### Immunohistochemistry

Formalin-fixed, paraffin-embedded tissue sections (4 µm) were de-waxed in xylene and rehydrated through graded alcohols to water. Endogenous peroxidase was blocked using 1.6% H_2_O_2_ for 20 min. For antigen retrieval, sections were pressure cooked in 10 mmol/l citrate buffer (pH 6.0) for 5 min. Sections were blocked with 10% serum for 30 min. Slides were incubated with primary antibody anti-rat Ki-67 TEC-3 (DAKO, Glostrup, Denmark; 1 : 125 dilution) for 1 h. Goat anti-rabbit secondary antibody was applied for 1 h at room temperature. Sections were then incubated in ABC (Vector Labs, Burlingame, CA, USA) for 30 min. DAB solution was applied for 2–5 min and development of the colour reaction was monitored microscopically. Slides were counterstained with haematoxylin, dehydrated, cleared, and then mounted. Images were taken of the E18.5 lung Ki67 immunohistochemistry at 20× magnification. For each sample, images of two separate areas of tissue were taken and then randomly within these images, three equally sized sections were identified. Counts were performed of the total number of cells and the number of Ki67-positive cells within these sections.

### Western blotting

Tissue samples were homogenized in Mega9 or RIPA lysis buffer with the addition of protease inhibitors (Complete Mini, Roche). Cells were lysed on ice for 20 min. Nuclear fractions were generated using lysis buffer A (Hepes 1000 mm, KCl 1000 mm, EDTA 1000 mm, DTT 1 mm, PMSF, NP40 10%, protease inhibitor) and centrifuging for 5 min. The pellets were then resuspended and lysed with buffer B (Hepes 20 mm, NaCl 400 mm, EDTA 1 mm, glycerol 10%, DTT 1 mm, PMSF, protease inhibitor), and agitated at 4 °C for 2 h. The samples were spun and the supernatant represented the nuclear fraction. All lysates were quantified using the BCA assay (Thermo Fisher Scientific, Waltham, MA, USA). Western blotting was carried out with the NuPAGE Gel system (Invitrogen Carlsbad, CA, USA) according to the manufacturer's protocol. Denatured lysates were loaded onto a 4–12% gel and run at 100 V for at least 2 h. The gels were transferred onto PVDF membrane (Immobilon P, Millipore, Billerica, MA, USA) in a semi-dry tank (120 mA for at least 2 h) and blocked by incubating for 1 h at room temperature in PBS or TBS containing 5% milk (Marvel).

The membranes were then incubated overnight in the appropriate primary antibody in TBS with 5% milk, anti-rabbit Klf5 (ab24331; Abcam, Cambridge, UK; 1 : 250 dilution), and anti-rabbit Tgif (H-171) (sc-9084; Abcam; 1 : 200 dilution). Lamin B1 (H-90 sc-20 682; Santa Cruz Biotechnology, Santa Cruz, CA, USA; 1 : 1000 dilution) was used as a loading control for nuclear fractions. After washing, the membranes were incubated with HRP-conjugated goat anti-rabbit secondary antibody and the HRP-conjugated anti-beta actin antibody (Abcam 8226; 1 : 10 000 dilution, as loading control) for 1 h at room temperature. Blots were incubated in ECL reagents (GE Healthcare, Buckinghamshire, UK) and detected by chemiluminescence film (GE Healthcare). To quantify the bands, an area which included each band was selected and the pixels were counted (ImageJ); the same area was used for each sample. After taking into account the value for background, the pixel value was normalized to its loading control.

## Results

### Arginine residues in the WD40 region of FBXW7 are conserved between human and mouse

**Figure 1:**
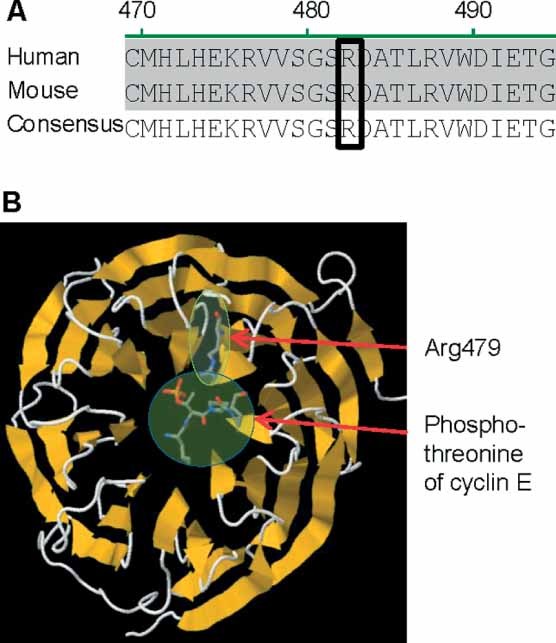
Fbxw7 alignment and molecular structure. (A) Alignment of FBXW7 protein sequence of human and mouse. The boxed region shows an arginine residue (human Arg479, mouse Arg482) in the WD40 domain which is commonly mutated in cancer patients. This residue is conserved between mouse and human FBXW7 and thus allows the generation of an informative mouse model. (B) Molecular model of FBXW7 WD40 propeller domain showing the effect of the human Arg479 mutation on the substrate binding site. The targeted arginine (human Arg479) is at the apex of one of the beta-sheets and interacts with the phosphothreonine of CCNE1 (an FBXW7 substrate). This interaction is predicted to be abolished if the arginine is mutated to glutamine. This protein model was generated using RasMol software

Approximately half of all cancer-associated *FBXW7* mutations occur at two hotspots, arginine residues at codons 465 and 479 within the WD40 repeats 10. We aligned the FBXW7 human and mouse protein sequences and confirmed that the arginine at position 479 is present in mice (at codon 482) and lies within a conserved region (Figure 1A). A molecular structure model confirmed that the R482Q mutation is at the apex of one of the beta sheets in the WD40 domain and thus is critical in substrate recognition (Figure 1B). Two *in silico* prediction programs, SIFT and Polyphen (http://sift.jcvi.org/, http://genetics.bwh.harvard.edu/pph/), predicted that R479Q substitution, from a basic to an uncharged polar amino acid, affects protein function and may be damaging.

### Generation of R482Q mice

**Figure 2:**
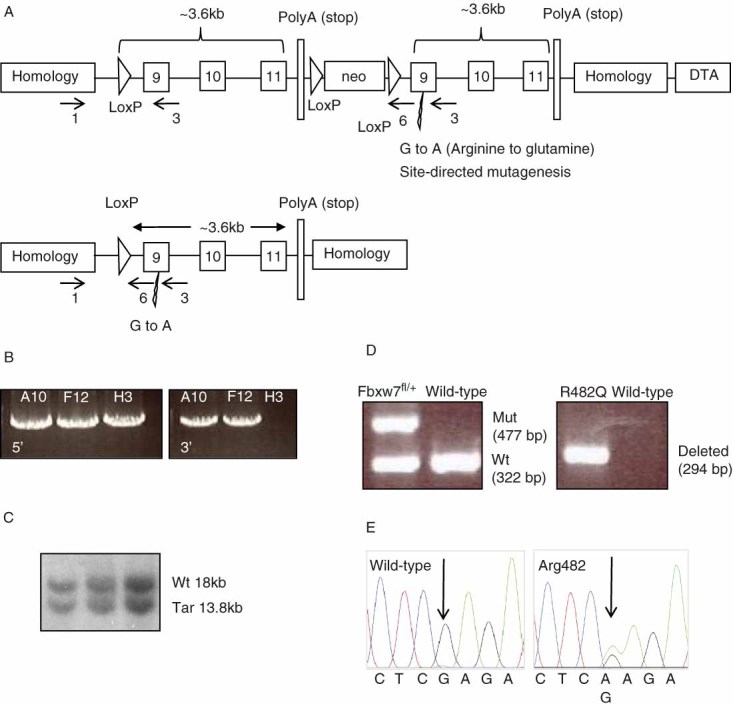
Generation of Fbxw7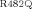 mouse model. (A) Schematic diagram of targeting scheme to generate Fbxw7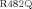 mouse model. The upper panel shows the targeting construct with regions of homology; loxP sites; genomic Fbxw7 exons 9, 10, 11; a neomycin selection cassette; and repeated exons 9, 10, 11 in which a mutation in exon 9 has been introduced by site-directed mutagenesis. The lower panel represents the allele after Cre-mediated recombination. The arrows with corresponding numbers depict the position and direction of the primers used to genotype the R482Q mice. (B) Long-range PCR genotyping of electroporated ES cell clones showing clones with positive 5′ (A10, F12, H3) and 3′ (A10, F12) integration. The band sizes of the 5′ and 3′ PCR products are 5371 and 5533 base pairs, respectively. (C) Southern blot screening showing wild-type and targeted bands (see the Materials and methods section). (D) Genotyping PCR screening of R482Q mice. The left panel shows the PCR product using primer pair 1 and 3 to identify the R482Q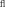 and wild-type alleles. The right panel shows the PCR product using primer pair 1 and 6 to identify the R482Q allele. (E) Sequence traces showing heterozygous point mutation in R482Q mice

Figure 2A shows the targeting construct used to generate our *Fbxw7*-mutant mouse. The targeting construct contained a loxP site upstream of exon 9 and two other loxP sites, flanking a Neo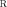 cassette, downstream of exon 11. On the other side of the distal loxP, we inserted a repeated genomic region containing exons 9–11, but with an R482Q allele created by site-directed mutagenesis within exon 9. We anticipated that Cre-mediated recombination would generally remove the genomic sequence between the most proximal and distal loxP sites, removing the NeoR cassette and endogenous exons 9–11 and allowing the transcription of the ‘knock-in’ mutated exon 9; in all the mice subsequently reported in this study, recombination had occurred between these outer loxP sites.

The targeting construct was electroporated into embryonic stem (ES) cells and screened for homologous recombination. Long-range PCR was initially used to screen for 5′ and 3′ integration (Figure 2B). Southern blotting was used to confirm the positive clones (Figure 2C), one of which was selected for subsequent introduction into mouse blastocysts to generate a chimera. Germline transmission occurred and the offspring were continuously back-crossed onto a C57Bl/6J genetic background. Mice carrying ‘floxed’ R482Q alleles were identified by PCR-based genotyping (Figure 2D). No abnormal phenotype was seen in the *Fbxw7*^*flox*/+^ and *Fbxw7*^*flox*/*flox*^ animals up to 13 months of age.

To investigate the effect of a germline *Fbxw7* mutation, we attempted to generate a constitutive mutant *Fbxw7*^*R*482*Q*/+^ (R482Q) mouse by crossing *Fbxw7*^*flox*/+^ animals with ubiquitously expressing Cre mice (Pgk Cre) 22. However, none of the offspring of these crosses (*N* = 74) carried the R482Q allele. We collected embryos at various stages of development and observed the expected 1 : 1 Mendelian ratios of heterozygous (R482Q; Neo^−^) and wild-type embryos up to E18.5 (*p* = 0.98, χ^2^ test) (Figure 2E). No striking differences were found between the mutant and the wild-type embryos with regard to gross external appearance or weight. We concluded that the embryos were probably dying at birth and confirmed this by observation of dead pups shortly after birth. It was, of course, impossible to generate homozygous mutants, due to the lethality in the heterozygous state.

To date, there is not an efficient antibody available to analyse Fbxw7 at a protein level but we found no significant difference in the level of *Fbxw7* mRNA expression between R482Q and wild-type E13.5 embryos (Supporting information, Supplementary Figure 1A). Nearby genes were analysed for disruption and no significant differences were observed (Supporting information, Supplementary Figure 1B).

Various tissues were dissected from E18.5 embryos and the expression of *Fbxw7* mRNA was analysed by real-time Q-PCR. *Fbxw7* was most highly expressed in the brain and the lung (Supporting information, Supplementary Figure 2), and there were no significant differences in expression between R482Q and wild-type mice in any of the tissues analysed. In the lung, the most abundant *Fbxw7* species present was the alpha isoform (data not shown).

### R482Q embryos have thickened alveolar septa in their lungs

**Figure 3:**
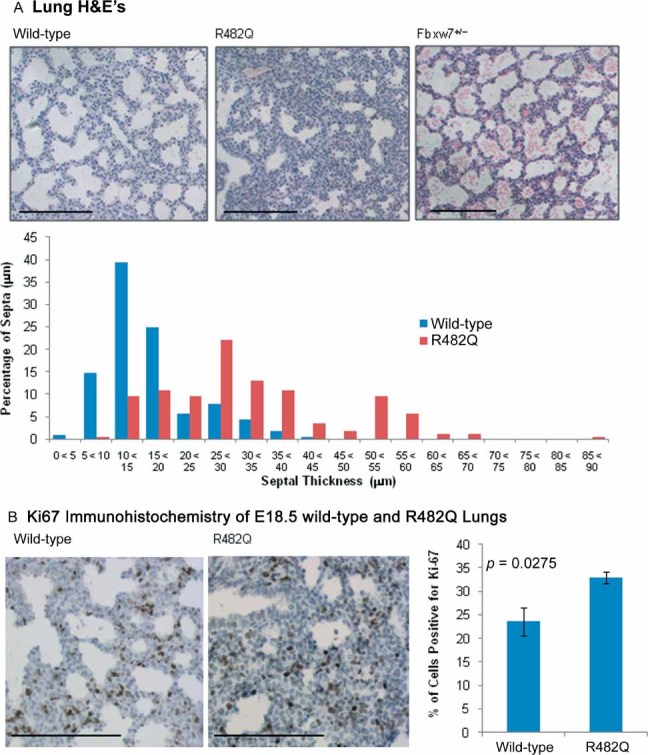
Phenotype of R482Q and Fbxw7^+/−^ E18.5 lungs. (A) H&E staining on E18.5 lung samples. Left panel: wild-type; middle panel: R482Q; right panel: Fbxw7 null heterozygotes (Fbxw7^+/−^). Scale bars are 150 µm. The R482Q lungs have thickened alveolar septa, whereas the wild-type and Fbxw7^+/−^ have normal-thickness alveolar septa. The graph shows a summary of the septal thickness in wild-type and R482Q E18.5 lungs. (B) Ki67 immunohistochemistry staining of wild-type (left panel) and R482Q (middle panel) E18.5 lungs. Scale bars are 150 µm. There is an increase in the percentage of Ki67-positive proliferating cells in the R482Q sample shown in the graph in the right panel (*p* = 0.0275, *t*-test). Error bars represent the SEM

We analysed E18.5 R482Q embryos for physiological and histological abnormalities to explain the perinatal lethality and noted a striking difference in the histology of the embryonic lungs (Figure 3A). R482Q embryos had thickened alveolar septa in comparison to their wild-type littermates. Wild-type lung septa had a mean width of 16.5 µm (range 2.8–41.7 µm) compared with 32.5 µm (range 8.3–88.9 µm) in R482Q animals (*N* = 32, *p* = 1.05 × 10^−11^, *t*-test).

We also examined lungs at an earlier stage of embryonic development E16.5 (Supporting information, Supplementary Figure 3). At this stage, which is before thinning down of the septum begins, there was no difference apparent between R482Q and wild-type lungs. We concluded that the pseudo-glandular stage of lung development (E9.5–E16.6) proceeded normally in the R482Q animals, but there was disruption of the canalicular (E16.6–E17.6) and/or terminal sac stage (E17.6–P5).

We compared our *Fbxw7*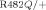 animals with Fbxw7-null mice bred in the same location in identical conditions and back-crossed to the same C57/BL6J stock 4. The heterozygous null (*Fbxw7*^+/−^) animals showed no thickening of lung septa or other lung abnormality (Figure 3A), showing that the effect that we observed in our R482Q mice was not the result of Fbxw7 haploinsufficiency. Homozygous null embryos could not be analysed as they died too early for assessment of lung development, but our mice no showed no cardiovascular abnormalities similar to those found in *Fbxw7*^−/−^ embryos. Overall, the functional consequences of the R482Q mutation differ from those of null homozygotes or heterozygotes and are arguably intermediate in their severity; in the developmental context, R482Q cannot be acting in a strict dominant negative or formally haploinsufficient manner.

### Other developmental abnormalities in R482Q embryos

**Figure 4:**
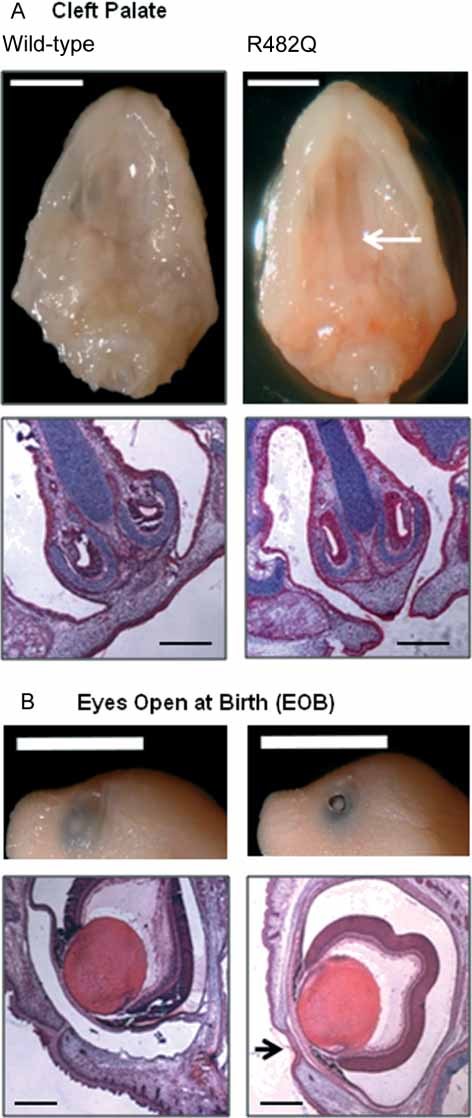
Other developmental abnormalities. (A) Cleft palate. The upper left panel shows the gross appearance of wild-type E18.5 palate. The upper right panel shows the gross appearance of the R482Q palate, with the arrow highlighting the position of the cleft palate. The scale bar is 0.5 cm. The lower panels (left, wild-type; right, R482Q) show H&E-stained sections; the scale bar is 300 µm. (B) Eyes open at birth (EOB). The upper left panel shows the gross appearance of normal eyelid fusion in wild-type E18.5 embryos. The upper right panel shows the EOB phenotype in E18.5 R482Q embryos; the scale bar is 0.5 cm. The lower panels (left, wild-type; right, R482Q) show H&E-stained sections; the scale bar is 300 µm. The arrow highlights the failure in eyelid fusion in R482Q

Perinatal lung developmental defects are often associated with disruption of the Tgf-β pathway, and we therefore checked our embryos for other phenotypes characteristic of Tgf-β defects. Thirty per cent (12/40) of the R482Q embryos had a cleft palate (Figure 4A). 42.5% (17/40) of the R482Q embryos had an ‘eyes-open-at-birth’ (EOB) phenotype (Figure 4B). Sixty per cent (24/40) of the R482Q embryos showed these other developmental phenotypes (one phenotype 47.5%, 19/40; both phenotypes 12.5%, 5/40). Neither the cleft palate nor the EOB phenotype was present in wild-type mice. During mammalian development, there is temporary fusion of the eyelids; the eyelids start to form at E11.5, grow across the eye from E14, and eyelid fusion is complete by E16 23. In our mutant mice, the eyelids did not reach each other to fuse. Palatogenesis is a multi-step process involving palatal shelf growth downward from the maxillary processes, elevation above the tongue, and fusion of palatal shelves. Murine palatogenesis takes places between E12.5 and E15.5 24. Defects in any aspect of this process can lead to a cleft palate.

### Molecular analysis of R482Q E18.5 lungs

Ki67 immunohistochemistry showed that there was a significantly increased level of proliferation in the R482Q E18.5 lungs compared with wild-type animals (Figure 3B). Very few lung cells had undergone apoptosis, as indicated by caspase 3 immunohistochemistry (see Supplementary Figure 4A in the Supporting information). No differences were observed in the location or proportion of specific lung cell lineages, specifically using CC10 for Clara cells (Supporting information, Supplementary Figure 4B) or proSP-C for alveolar type II cells (Supporting information, Supplementary Figure 4C).

**Figure 5:**
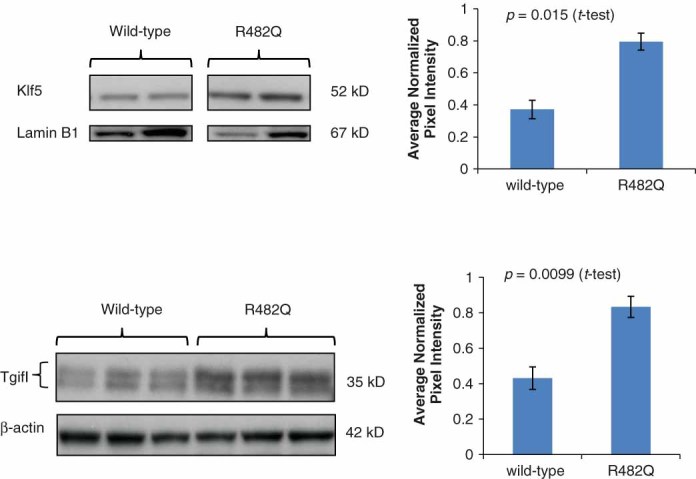
Tgf-β signalling components western blots on E18.5 lungs. The upper panel shows the increased level of Klf5 in nuclear fraction R482Q E18.5 lung samples; lamin B1 was used as a nuclear fraction loading control. The upper graph shows the average normalized pixel intensity. The lower panel shows the increased level of Tgif1 in R482Q E18.5 lung samples. The upper Tgif1 band represents the hyperphosphorylated Tgif1 and the lower band the hypophosphorylated Tgif1. β-actin was used as a loading control. The lower graph shows the average normalized pixel intensity. To generate this graph, both hyper- and hypo-phoshorylated bands were quantified together. There is also a significant difference in the average pixel intensity between the hyper- and the hypo-phosphorylated bands when quantified independently (*p* = 0.0060, *p* = 0.0229, *t*-test, respectively). Error bars represent the SEM

We wished to determine which of the numerous protein targets of Fbxw7 were differentially expressed and therefore carried out western blot analysis on E18.5 lungs. The proteins initially analysed were Aurka, Ccne1, Nicd1 (active Notch1), Jun, and Myc (Supporting information, Supplementary Figure 5). Surprisingly, these well-known Fbxw7 targets showed no difference between R482Q and wild-type lungs. However, we found that two more recently identified substrates of Fbxw7, Klf5 25 and Tgif1 26, were present at increased levels in R482Q lungs (Figure 5). We confirmed that there were no differences in the mRNA expression of *Tgif1* and *Klf5*, as would be expected if effects were mediated by Fbxw7 (Supporting information, Supplementary Figure 6). Tgif1 protein was also increased in R482Q mouse embryonic fibroblasts (MEFs) in comparison to wild-type MEFs (Supporting information, Supplementary Figure 7). Klf5 is not expressed until later stages of embryonic development, so cannot be detected in MEFs 27.

## Discussion

Using our R482Q mouse model, we have shown that specific WD40 missense mutations have a different effect from Fbxw7 heterozygous null or homozygous null mutations. Our R482Q mutant Fbxw7 affects embryonic epithelial development. A combination of defects in lung maturation, palatogenesis, and eyelid fusion caused lethality in heterozygotes within hours of birth. The cause of death was almost certainly the lung phenotype, as EOB is not life-threatening and cleft palate does not usually lead to death this early. The relatively high levels of expression of *Fbxw7* in the developing lungs suggest a particularly important role in this organ and may explain the predominance of the lung phenotype in R482Q embryos. By contrast, the cleft palate and eyelid fusion phenotypes were incompletely penetrant.

*Fbxw7* homozygous null mice display earlier embryonic lethality at E12.5 due to compromised vasculogenesis 17, 18, the heterozygotes surviving to adulthood with no apparent defect. Disruption of Notch was considered the primary contributor to the phenotype of homozygous null animals. We neither found the level of Notch protein to be elevated in the lungs of our mice, nor observed effects on Jun levels previously found when we targeted *Fbxw7* inactivation to the gut 4. Conversely, since our epithelial phenotypes are not observed in the heterozygous *Fbxw7* null models, these must be the direct result of the specific missense R482Q mutation. It is of note that all *Fbxw7* models discussed here are on a C57Bl6J background.

Murine lung development can be described in four phases 28. Lung development commences at embryonic day (E) 9.5, firstly with the pseudo-glandular phase, during which the bronchial and respiratory tree develops to form an undifferentiated system. The canalicular phase, from E16.6 to E17.4, compromises terminal lung bud dilation and vascularization. The saccular phase, from E17.4 to post-partum day (P) 5, includes the formation of distal airways sacs, the thinning down of the mesenchyme layer, continuation of vascularization, and differentiation of pneumocytes. Finally, the terminal sacs develop into mature alveolar ducts and alveoli by P30. There are several mouse models which display lung developmental phenotypes. For example, deletions of *C/EBPα, Foxa2*, and *Nfib* result in perinatal respiratory distress due to incomplete lung development 29–32. Mutation in *Tgf-b3* results in delayed lung development, with an additional cleft palate phenotype 33. Additionally, an *Fbxo11* mutant causes an increase in phospho-Smad2 which results in delayed lung development, a cleft palate, and an EOB phenotype 34.

Whilst most substrates of Fbxw7 remained unaltered in R482Q lungs, we showed a significant increase in Klf5 and Tgif1. Both Klf5 and Tgif1 are involved in multiple pathways, several of which are known to be important in lung development.

Tgif1 knockdown *in vitro* causes disruption of both Tgf-β and retinoic acid pathways 35. Tgif1 is a transcriptional repressor that negatively regulates the Tgf-β pathway via several mechanisms. Tgif1 acts as a repressor by recruiting specific repressor complexes to Smad2 to repress Smad2-mediated transcription. It regulates a number of Tgf-β-dependent processes, including transcription, proliferation, and cell migration. It is interesting to note that Tgif1 also interacts with c-Jun, another target of Fbxw7 36. This interaction has been shown to be important for the ability of Tgif1 to repress Smad-dependent transcription.

Klf5 is a zinc finger domain-containing transcription factor that plays roles in *Tgf-b*, retinoic acid, and PKC pathways, among others 37. To date, hundreds of genes regulated by Klf5 have been identified, associated with processes including cell cycle regulation, apoptosis, migration, differentiation, angiogenesis, and lipid metabolism 38, 39. Interestingly, one of the genome-wide microarray studies which identified Klf5 targets used a lung-specific *Klf5* knockout mouse model 39. It is notable that *Klf5* conditional deletion inhibits lung maturation and is accompanied by an increase in *Tgf-b2* expression 39. *KLF5* is often considered an oncogene as it has many pro-proliferative effects 40, 41, and some of the important target genes are cyclin D1, cyclin B, PDGFα, and FGF-BP 37. However, there is evidence that in some cases, Klf5 may have an anti-proliferative effect; for example, when TGF-β is present, KLF5 acts as a co-factor to TGF-β signalling and exerts an inhibitory role on cell proliferation 42.

The reasons for the normal levels of other Fbxw7 substrates are unclear. However, several Fbxw7 substrates are regulated by more than one E3 ligase complex 43 and/or by other mechanisms which may occur in a tissue-specific manner. The relative abundance of the Fbxw7 isoform in different tissues may also play a role 44. Alternatively, and more intriguingly, our R482Q mutation may modify the binding of Fbxw7 to some substrates and not others. For instance, Klf5 and Tgif1 might be highly sensitive to the effects of this mutation.

Several studies have proposed that *Fbxw7* acts in a haploinsufficient manner in cancers and there is some evidence for this possibility: heterozygous null *Fbxw7* mutants have a greater susceptibility to radiation-induced tumourigenesis 2 and enhance intestinal tumourigenesis in an *Apc*-mutant background (although not when *Fbxw7* alone is mutated) 4.

However, in human cancers, the predominance of missense propeller tip mutations over protein-truncating mutations or other missense mutations argues against simple haploinsuffiency, and the data presented here clearly show functional differences between heterozygous null and R482Q alleles. Mutated Fbxw7 has also been proposed to act in a dominant negative fashion 10. Fbxw7 contains a dimerization domain near the N-terminus, and dimers may target specific substrates for degradation. Proteins containing missense mutations or non-sense mutations that leave the dimerization domain intact are still able to dimerize with wild-type Fbxw7. This reduces the number of active dimers and might be expected to have more detrimental effects than simple haploinsufficiency, comparable to the severe homozygous Fbxw7 null phenotype. However, our model has shown that R482Q is not a simple dominant negative. We are left with the possibility that this allele results in a level of protein function between those of the heterozygous and homozygous null states, or even different in some specific way in terms of substrate specificity or, whilst unlikely, gain of activity against non-physiological substrates.

We conclude that our *Fbxw7*^*R*482*Q*/+^ animals provide evidence that a ‘just right’ level of FBXW7 protein function is selected in many human cancers. In mouse development, this specific genetic abnormality does not cause vascular problems, but results in severe lung abnormalities caused not by changes in the well-established targets of FBXW7, but by increases in Klf5 and Tgif1.

## SUPPORTING INFORMATION ON THE INTERNET

The following supporting information may be found in the online version of this article.

**Figure S1A.** *Fbxw7* mRNA expression in wild-type and R482Q E13.5 whole embryos.

**Figure S1B.** Expression levels of genes surrounding *Fbxw7* in wild-type and R482Q.

**Figure S2.** Expression of *Fbxw7* in a range of tissue types in wild-type and R482Q E18.5 embryos.

**Figure S3.** Representative H&E-stained sections of wild-type and R482Q E16.5 lungs.

**Figure S4.** Caspase 3, CC10, and prosurfactant protein C (ProSP-C) immunohistochemistry of wild-type and R482Q E18.5 lungs.

**Figure S5.** Western blots for Fbxw7 substrates on E18.5 wild-type and R482Q lungs.

**Figure S6.** Expression of *Klf5* and *Tgif1* mRNA in E18.5 wild-type and R482Q lungs.

**Figure S7.** Western blots of Tgif1 protein expression in wild-type and R482Q MEFs.
