# Supplementary figures and images for: FBXW7 mutations typically found in human cancers are distinct from null alleles and disrupt lung development
Source: J Pathol. 2011 Apr 18;224(2):180–9. doi: 10.1002/path.2874 (PMC3757315; doi:10.1002/path.2874)

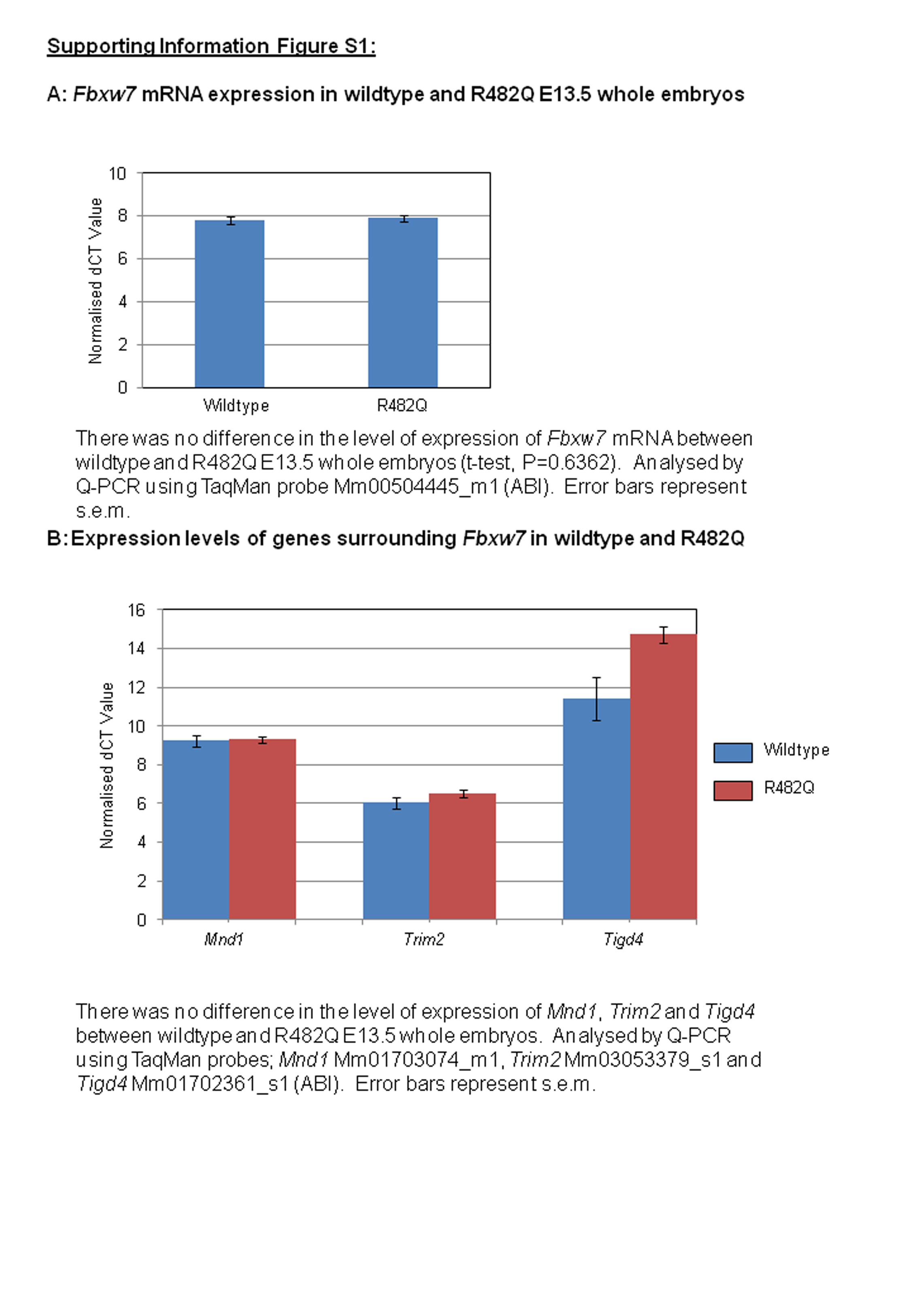

Supplement: Supplementary file 1 [file path0224-0180-SD1.tif]

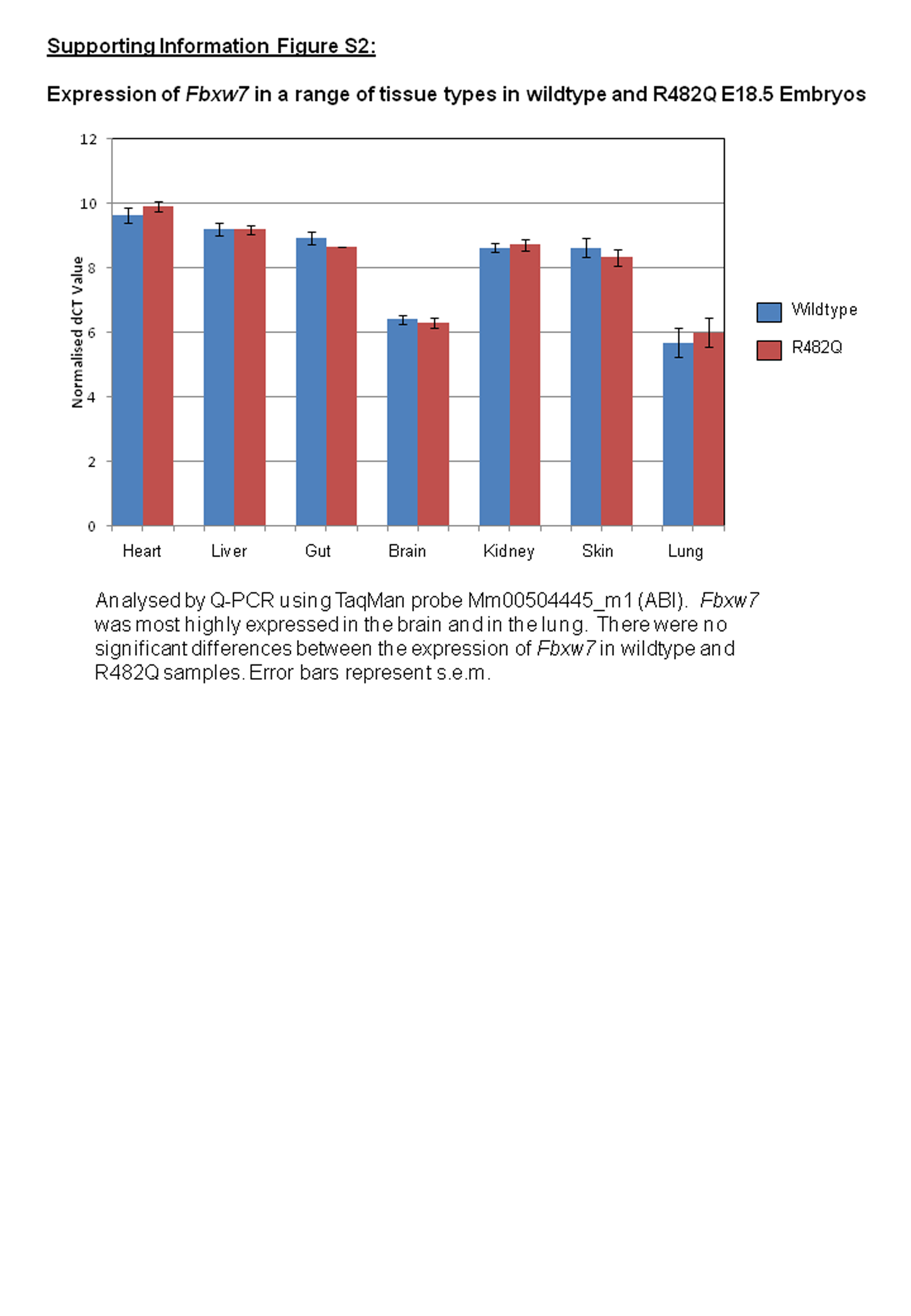

Supplement: Supplementary file 2 [file path0224-0180-SD2.tif]

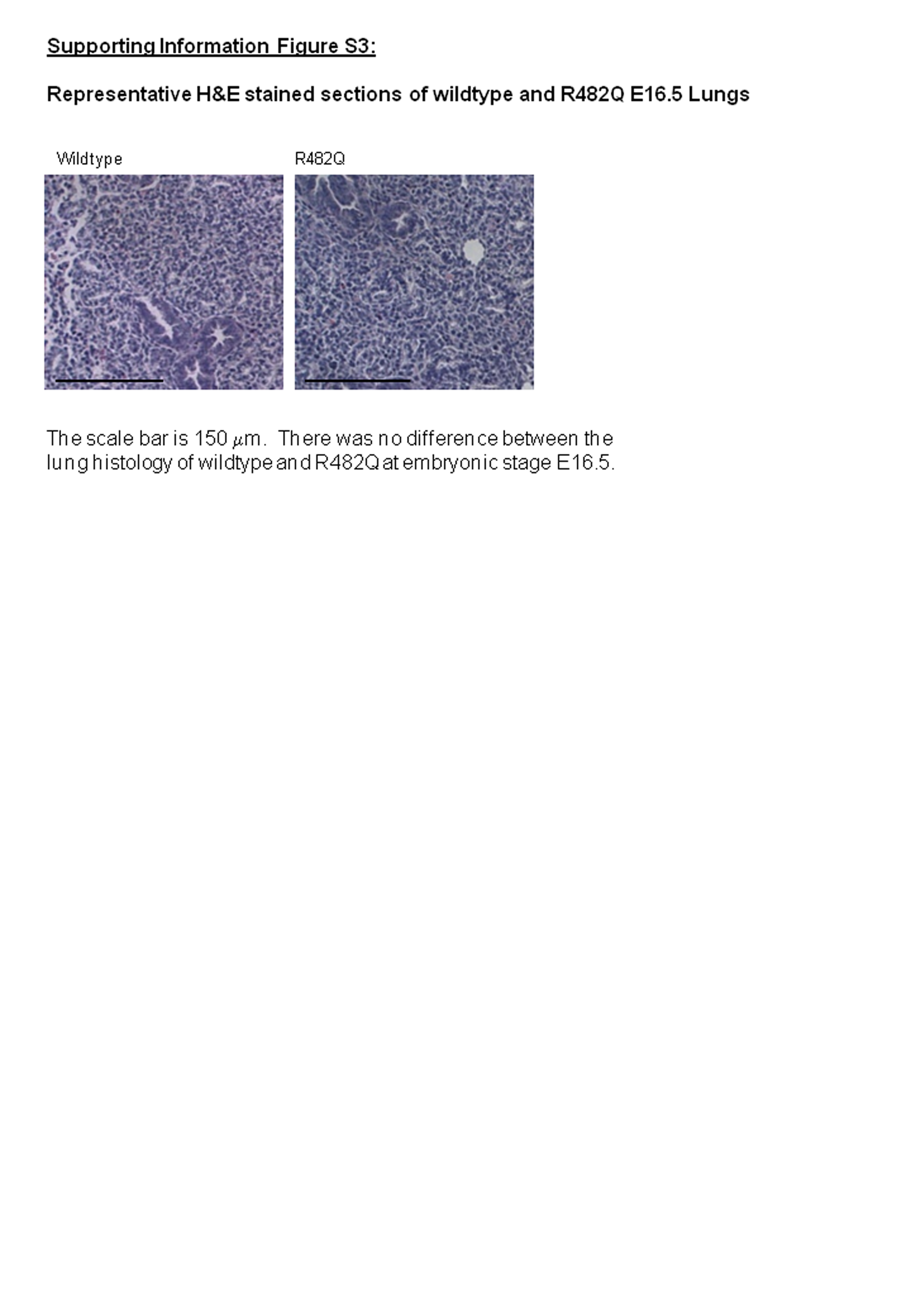

Supplement: Supplementary file 3 [file path0224-0180-SD3.tif]

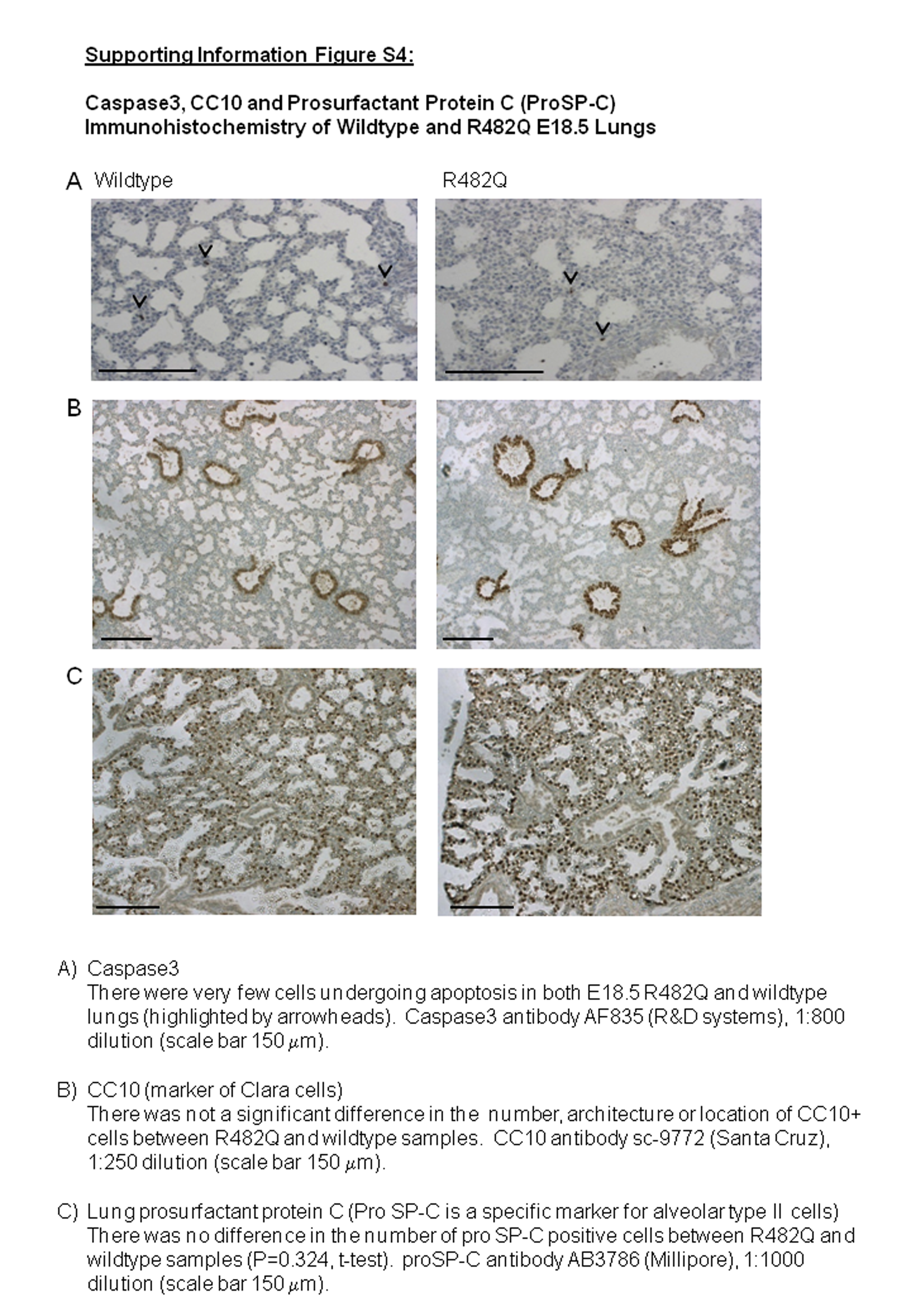

Supplement: Supplementary file 4 [file path0224-0180-SD4.tif]

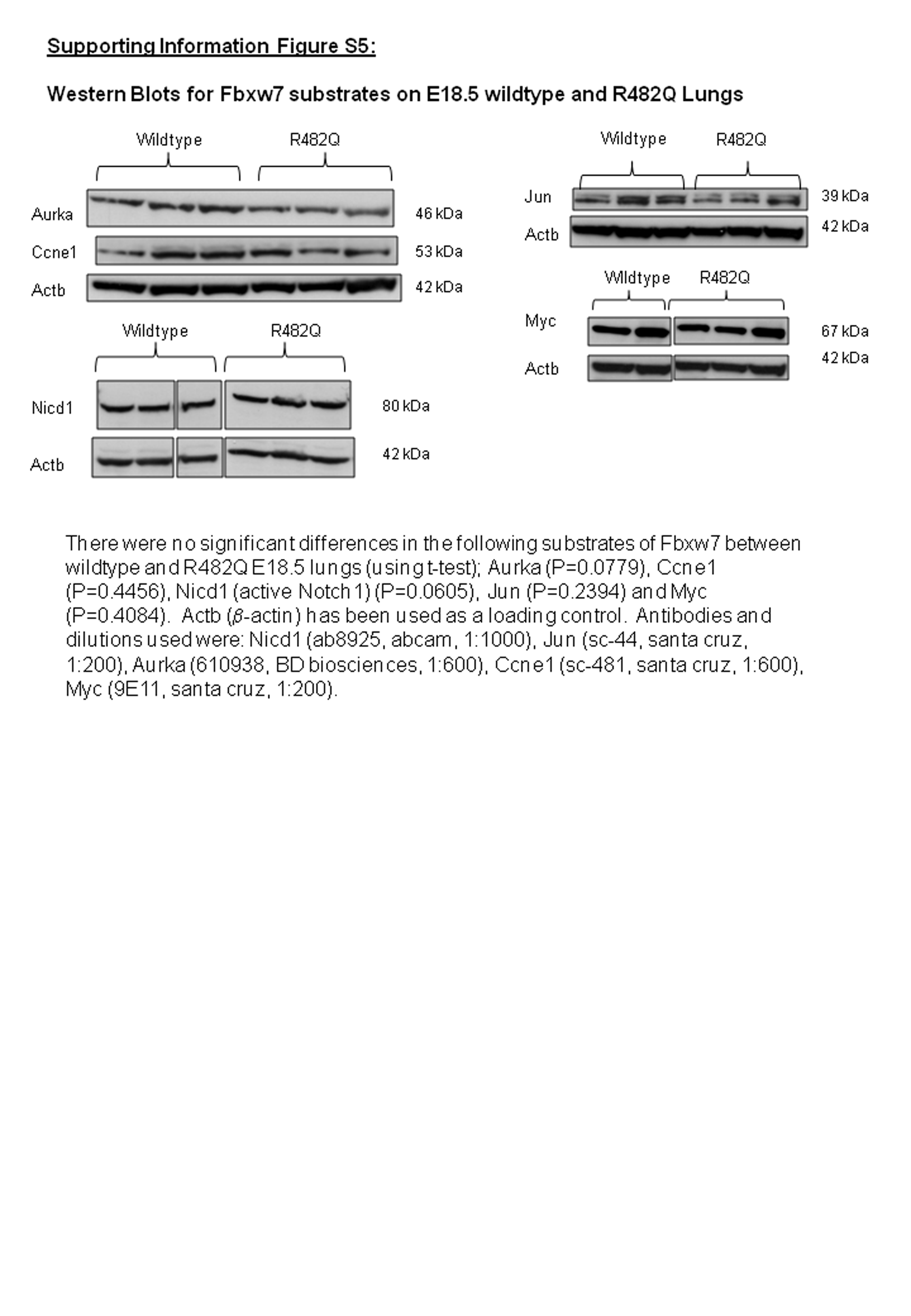

Supplement: Supplementary file 5 [file path0224-0180-SD5.tif]

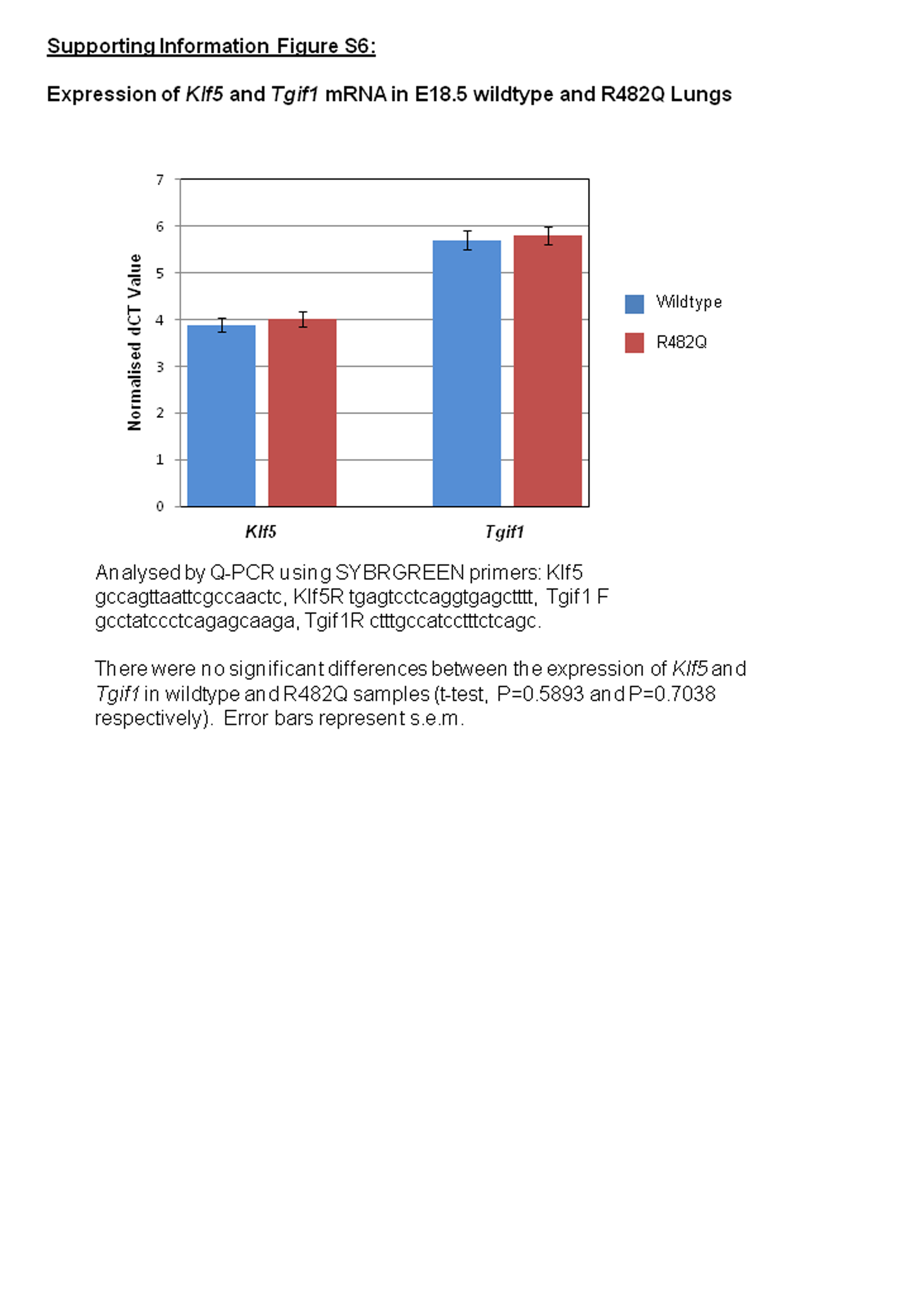

Supplement: Supplementary file 6 [file path0224-0180-SD6.tif]

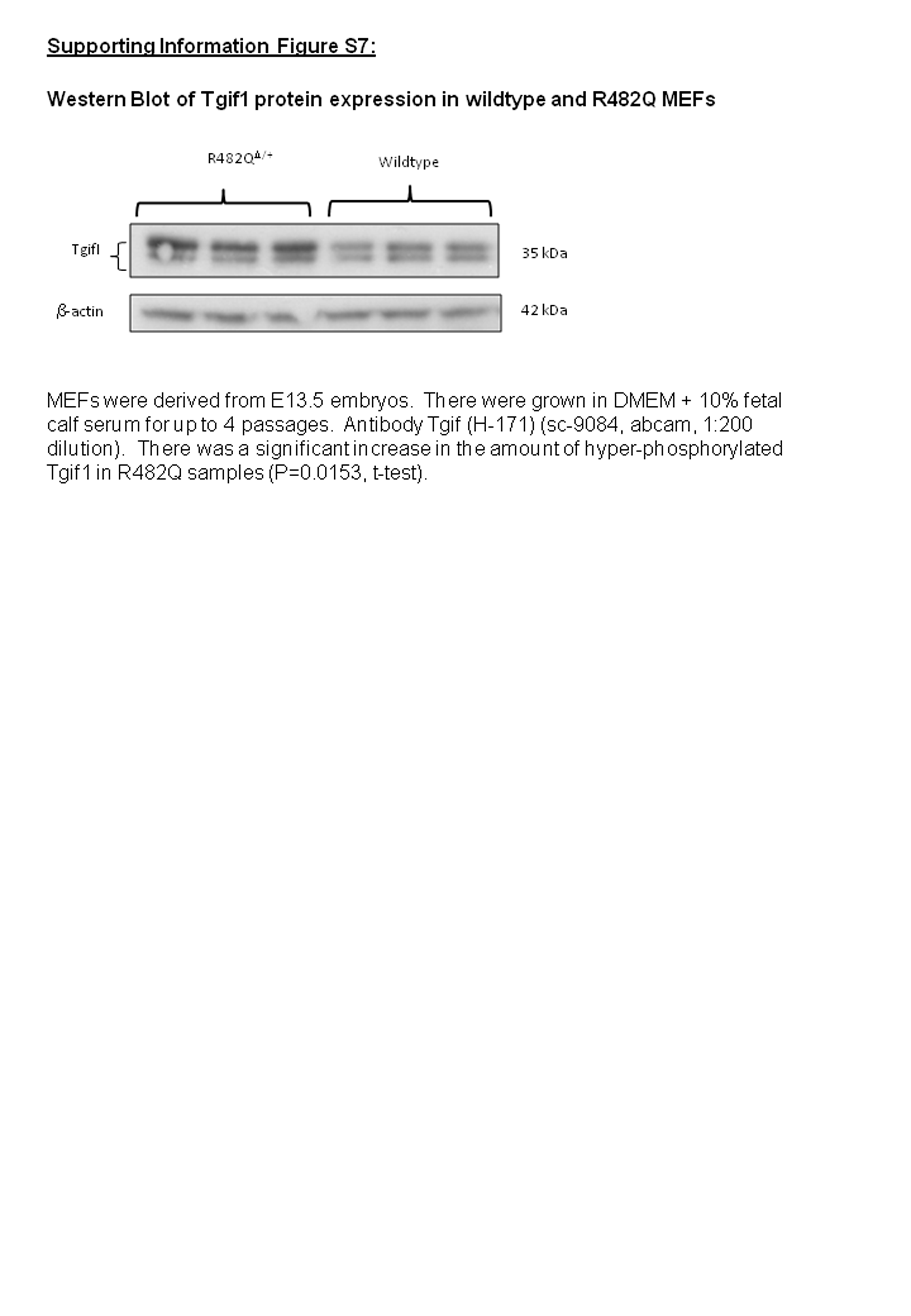

Supplement: Supplementary file 7 [file path0224-0180-SD7.tif]
